# Mesh Antibioma: A New Entity in the Presentation of Late-Onset Mesh Infection

**DOI:** 10.7759/cureus.36144

**Published:** 2023-03-14

**Authors:** Ashwin Jain, Chandrashekhar Mahakalkar, Suhas Jajoo, Chava Aravind Kumar

**Affiliations:** 1 Department of General Surgery, Jawaharlal Nehru Medical College, Datta Meghe Institute of Higher Education and Research, Wardha, IND

**Keywords:** antibioma, mature fibrous cyst, recurrent umbilical hernia, polypropylene mesh, deep mesh infection, ventral and incisional hernia, abdominal pseudocyst

## Abstract

Antibioma is a tough-walled abscess, which usually forms as a sequela of inadequate or lack of pus drainage during infection and inappropriate use of antibiotics by the patient. In this case report, we present a case of the development of antibioma due to infected polypropylene mesh used in umbilical hernia repair 10 years ago in a 59-year-old obese male. He had a history of umbilical and right inguinal hernioplasty 10 years back. Intraoperatively, we found antibioma whose wall was made up of fibrous mesh and the center was filled with pus and nonfibrous mesh remnants. The pus was found to be sterile, and the wall was made up of fibromuscular adipose tissue with chronic inflammatory cells around it. This is a very rare presentation of umbilical site deep mesh infection as it had no signs of acute inflammation, neither pain nor any pus discharge. We conclude that the possible explanation for the formation of antibioma and its very delayed presentation could be due to mesh infolding and seroma/hematoma formation during previous surgery may have led to the formation of abscess and thick fibrous wall without any fistulous tract and other complications of deep mesh infection.

## Introduction

Use of mesh for hernia repair dates back to 1989 when Lichenstein introduced tension-free mesh repair for lower recurrence rates and less postoperative pain [1]. One of the most dreaded complications of mesh usage is late-onset deep mesh infection, which was first defined by Mann in 1998 [2]. Postoperative complications of incisional hernia surgery such as seroma, hematoma, and abscess formation can lead to deep mesh infection. Symptoms of deep mesh infection include chronic pain, scar area tenderness, swelling, and redness. Some may present with pus discharging sinus/fistula [3,4]. The incidence of deep surgical site infection ranges from 0.3% to 0.5% [5].

Antibioma is a chronic tough fibrous abscess formed commonly due to prolonged and inappropriate use of antibiotics for infections. In this case report, the patient underwent umbilical hernia repair and an abscess was formed, but due to lack of pus drainage and lack of proper follow-up in the postoperative period with inappropriate use of antibiotics, he developed a sterile pus-containing antibioma, which was asymptomatic [6].

Another rare complication following mesh hernia surgery is a mature fibrous cyst, which was first described in 1993 by Waldrep et al. [7]. The term abdominal pseudocyst differs from antibioma in that aspect, it has thin walls, which are not lined by epithelium. They have an incidence of 0.88% in cases where mesh was used for hernia repair. When this pseudocyst wall becomes more fibrous, the term *mature fibrous cyst* has been used by many authors. Until now, only 18 cases of giant abdominal pseudocyst post mesh hernioplasty have been described in the literature, which develops within a mean period of one to 24 months from index surgery, and in all the cases, the cyst contained serous fluid [8-15]. 

We present a case report of late-onset deep mesh infection of umbilical hernia presenting as antibioma. The patient also had a recurrent umbilical hernia and epigastric hernia, 10 years after initial surgery, with no symptoms and signs of deep mesh infection and a positive history of obesity and smoking. This was a never seen or documented case of antibioma forming around a polypropylene mesh.

## Case presentation

Case history

**Figure 1:**
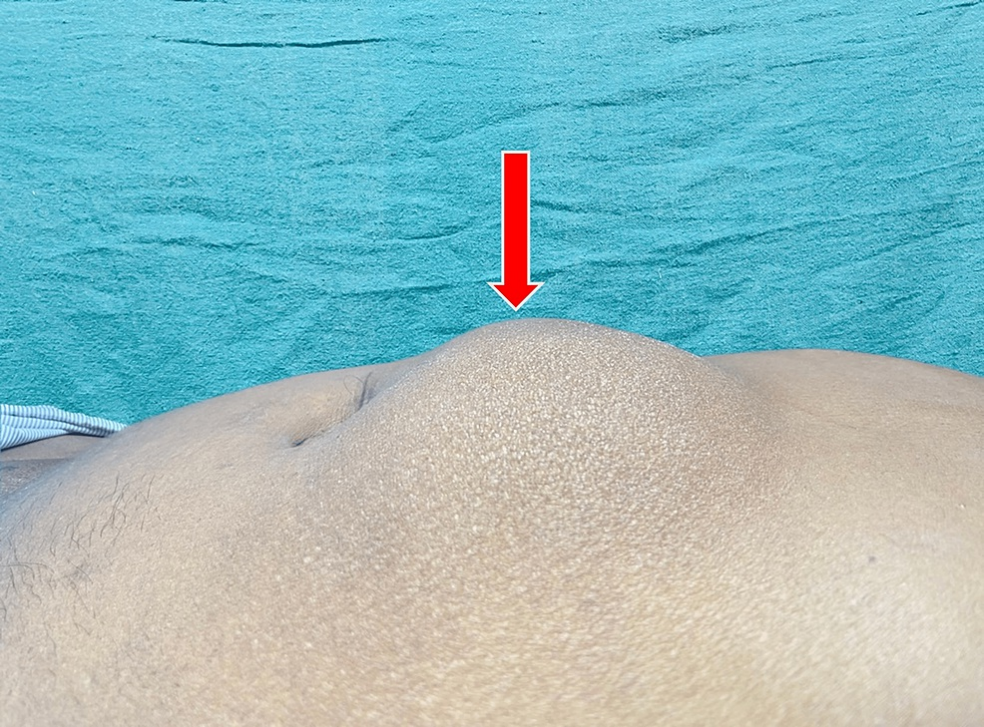
Red arrow showing an epigastric bulge.

**Figure 2:**
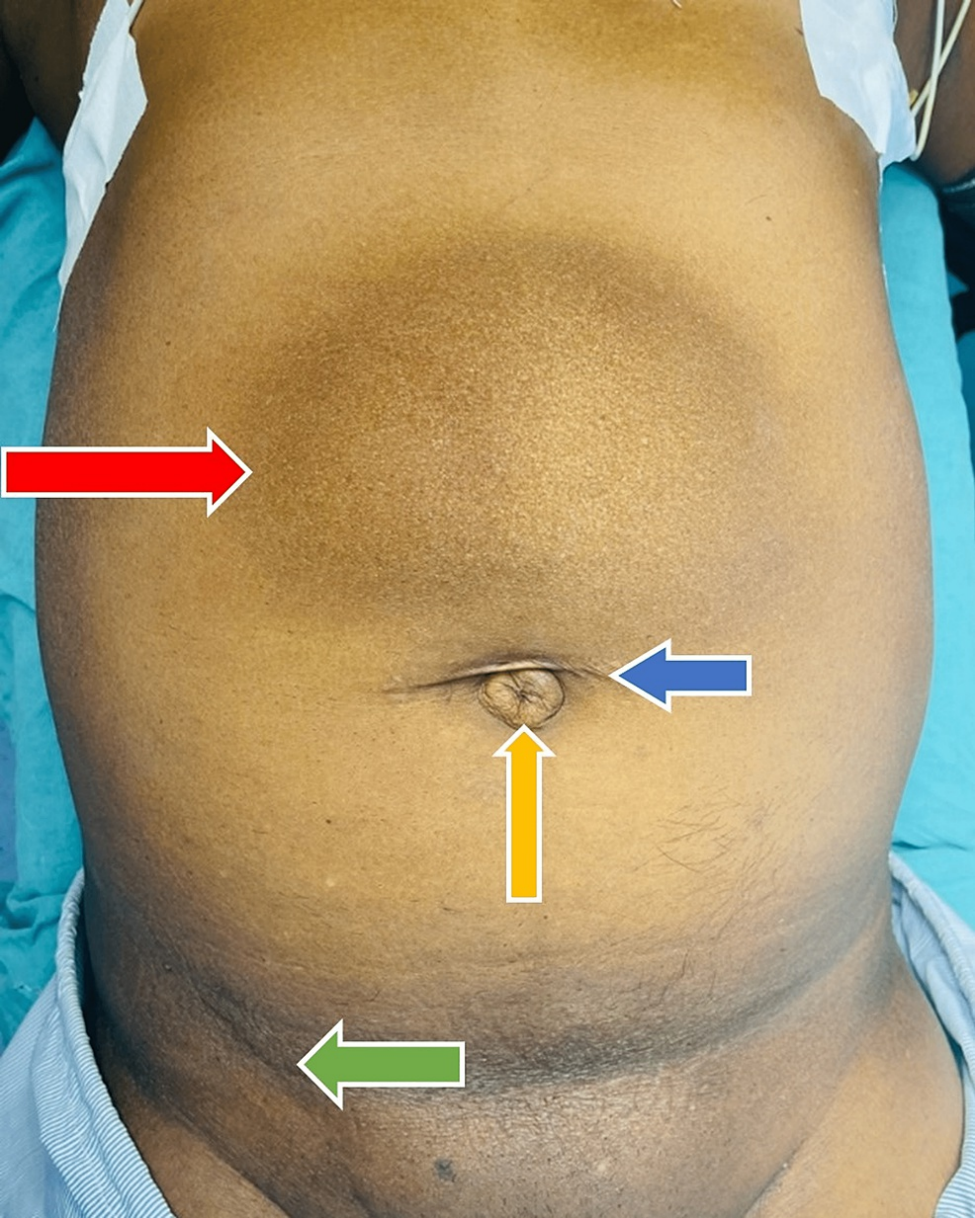
Red arrow showing a paraumbilical hernia, a blue arrow showing a scar mark of umbilical hernioplasty, a yellow arrow showing a recurrent umbilical hernia, and a green arrow showing a scar mark of the right inguinal hernia.

A 59-year-old male presented with a complaint of epigastric and recurrent umbilical swelling for two years; the swelling was insidious in onset and progressive in nature and increased on coughing and decreased on lying down, not associated with pain. The patient had been known to be hypertensive and on medication for the last four years and smoking tobacco for 12 years, which is associated with chronic cough. The patient has a history of umbilical hernioplasty and right inguinal hernioplasty 10 years back, but no documents are available. Following surgery, he has a history of taking a prolonged antibiotic and pain medication for postoperative pain, but no documents are available. The patient has no bowel and bladder complaints, and sleep and appetite are normal. On examination, there is 10 cm (horizontal) × 7 cm (vertical) swelling over the epigastric region in the midline with normal overlying skin and swelling becoming more prominent on head raising (Figures 1-2). Soft in consistency and cough impulse is positive, and no dilated veins over the swelling, with margins of the abdominal wall defect palpable circumferentially. There was a healthy transverse scar over the supraumbilical region measuring around 3 cm with no tenderness. A healthy scar at the right inguinal region was found. A defect of around 1 cm × 1 cm is located at the umbilicus with a positive cough impulse.

Management 

**Figure 3:**
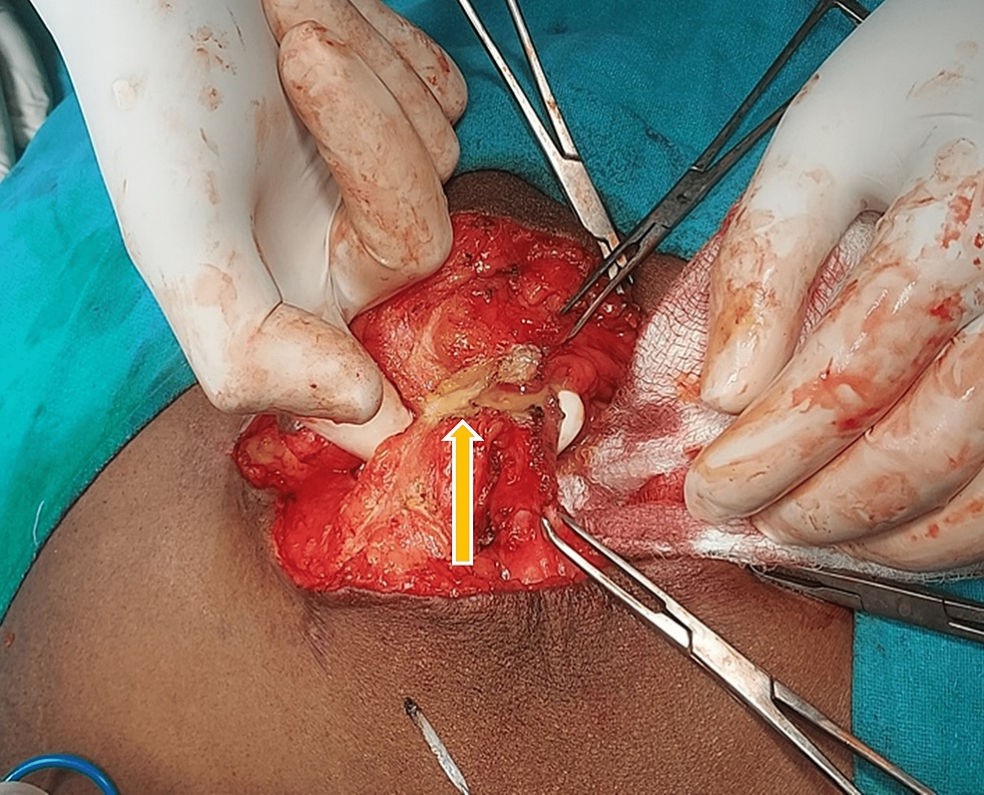
Yellow arrow showing pus discharge from the antibioma cavity draining out after incision.

**Figure 4:**
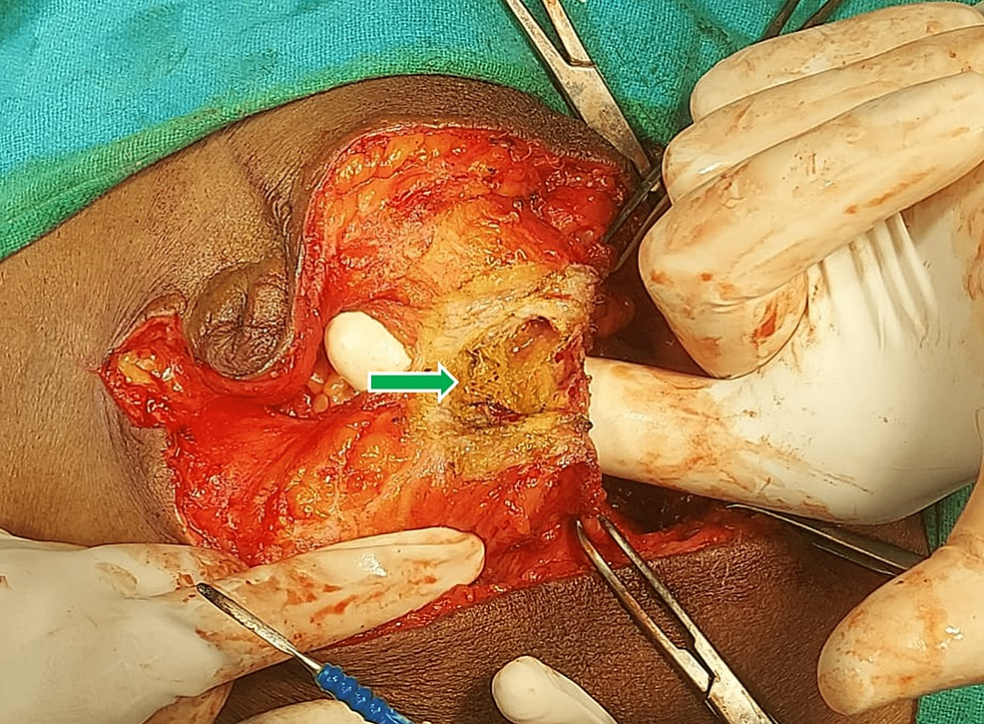
Green arrow showing an unfibrosed infected mesh at the center of the cavity.

**Figure 5:**
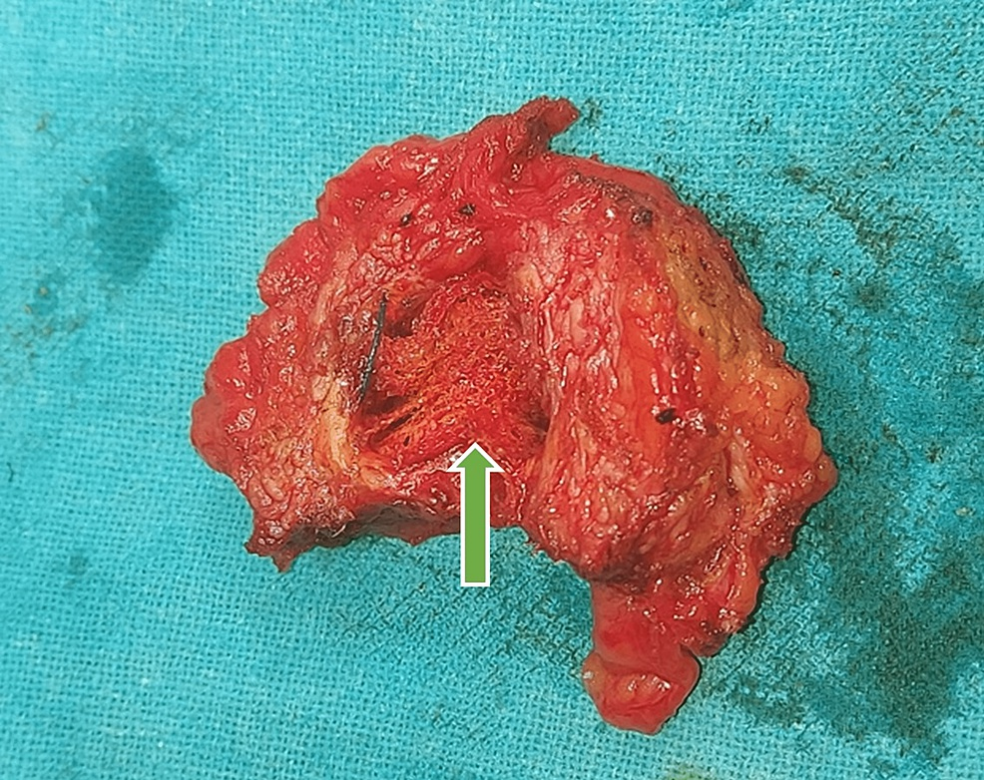
Arrow showing an infected mesh at the center of the exicised mesh antibioma.

Ultrasonography showed a defect of 10 cm × 8 cm in the epigastric region with bowel loops and omentum as content, a 1 cm × 1 cm defect at the umbilical region with omentum and fat as content, with grade 1 fatty liver. It also showed a cystic cavity of size 5 cm × 4 cm with hypoechoic fluid collection with moving internal echoes and thickened walls at the supraumbilical region with fat planes maintained all around. The patient was taken for surgery with the goal of complete excision of the cyst with hernia repair. Operative Findings are as follows. The supraumbilical hernia content was found to be transa verse colon, thick mature fibrous cyst of around 5 cm × 4 cm was found attached between both defects and was incised in the midline (Figures 3-4). After incision, a pus-filled cavity was found with active pus discharge of around 5 mL and polypropylene mesh as content; no fistulous tract was identified around the cyst, and the whole fibrous cyst with the wall was excised and removed (Figure 5). The pus on the culture was found to be sterile.

 

**Figure 6:**
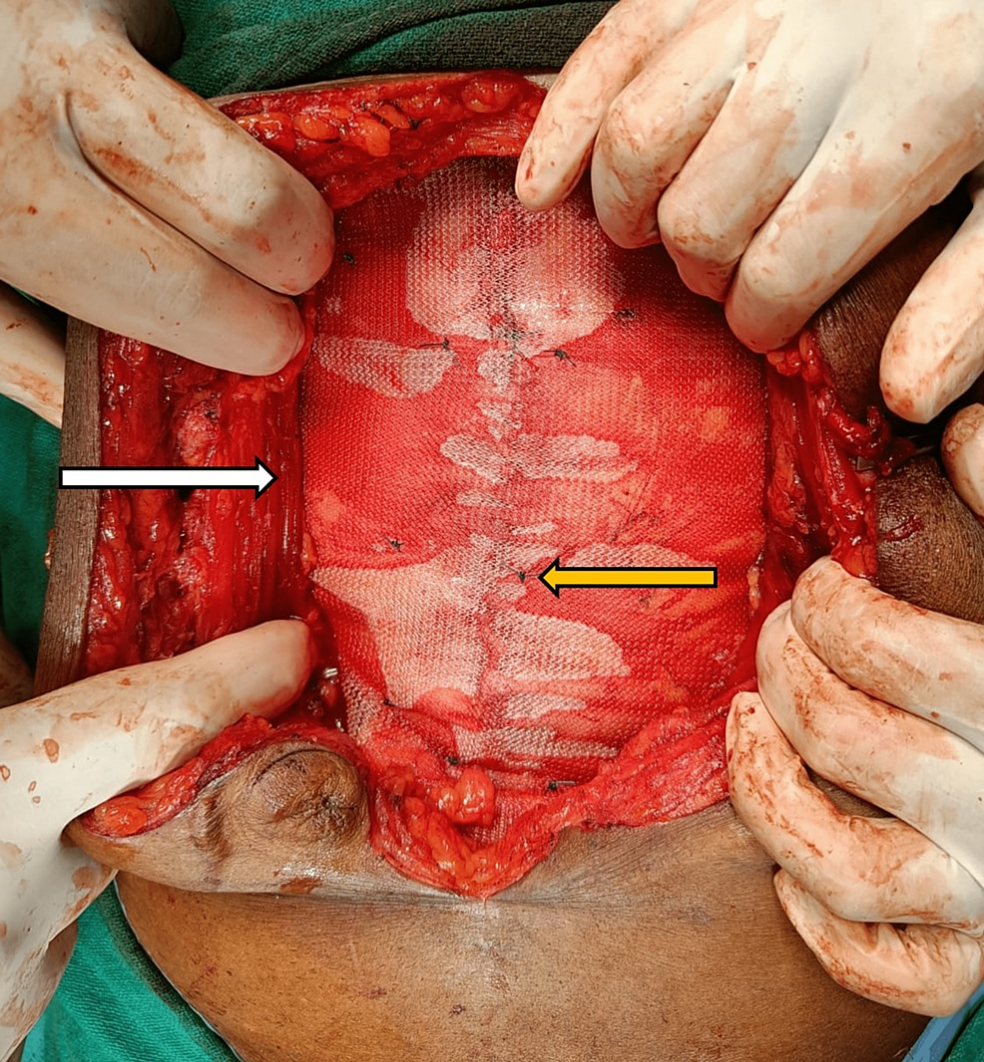
White arrow showing the retracted rectus muscle and mesh in the retrorectus plane without any folds and the yellow arrow showing mesh fixed to the posterior rectus sheath using Prolene 2-0 sutures.

Following the excision of the infected mesh, the posterior rectus sheath was separated from the linea alba from the xiphisternum above the arcuate line below and both layers were approximated using Prolene 2-0 (Ethicon/J&J, Raritan, NJ, USA) suture in a continuous interlocking manner. Polypropylene microporous synthetic mesh measuring 20 cm × 15 cm was kept in the retrorectus plane after a thorough saline wash and fixed to the posterior rectus sheath using interrupted sutures and the anterior rectus sheath closed using interrupted Prolene 1-0 suture (Figure 6). Two multiperforated suction drains were placed, one in the retrorectus plane and the other in the subcutaneous plane. The intraoperative and postoperative period was uneventful, and the patient was discharged with no complications.

## Discussion

**Figure 7:**
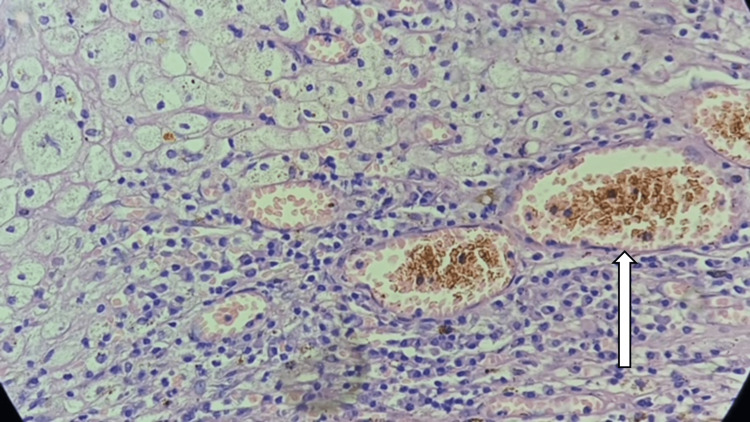
Histopathology slide of the fibromuscular adipose wall of antibioma, where the arrow represents the granulation tissue proliferated inside the mesh pore with surrounding histiocytes and chronic inflammatory cells.

**Figure 8:**
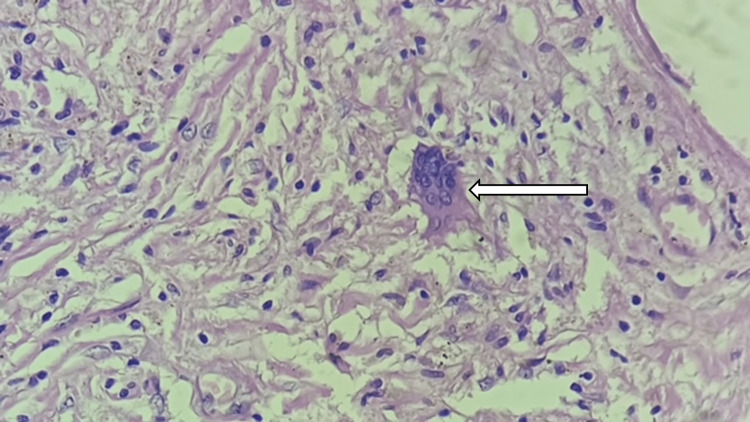
Arrow showing foreign body granuloma in the wall.

No case of mesh antibioma has been found in the literature until now; we have discovered a new presentation of deep mesh infection. Classically, deep mesh infection presents as acute inflammatory changes around the surgical region whose presentation can vary from months to years after surgery [4]. In the case aforementioned, we did not suspect deep mesh infection as the surgical scar was nontender, no overlying skin inflammatory signs were present, and no evidence of pus discharge or any fistulous opening and no bowel complaints were present. Intraoperatively, we discovered a fibrosed pus cavity with an unfibrosed mesh lying in the center, which was communicating to neither the overlying skin nor the underlying bowel, although the peritoneum has adhered to the fibrosed wall. The pus culture was sterile, and the histopathological slide revealed fibromuscular adipose tissue with central granulation with dense inflammatory infiltrate (Figure 7) and foreign body granulomas (Figure 8) surrounding the mesh representing a chronic inflammatory process leading to antibioma formation [6].

Antibioma can be confused with the diagnosis of a mature fibrous cyst or abdominal pseudocyst, which is a rare complication of hernia surgery, which follows seroma formation post mesh placement and usually presents at three to 24 months post index hernia surgery [7-15]. It is termed pseudocyst as it does not contain any epithelial lining. In this case, the presentation was 10 years after the index surgery and contained sterile purulent material. The decision was taken to excise the antibioma and do a retrorectus mesh placement as it has lower chances of recurrence and separate the mesh from the potentially infected subcutaneous and deep intraperitoneal space. The excised specimen revealed a fibrotic mesh in the wall and an unfibrosed part in the center, indicating that it could have been due to mesh folding onto itself at the time of placement or seroma/hematoma formation postoperatively. The patient had a history of tobacco smoking for 10 years and was obese, an important risk factor in developing deep mesh infection, thereby leading to antibioma [8,16].

## Conclusions

We conclude that late-onset deep mesh infection could also present silently as antibioma, without any acute inflammatory signs or fistulas. The mesh should be placed such as it is in full contact with the tissue, and no potential space should be left for seroma/hematoma/abscess formation. Sterile techniques to handle the mesh should be advocated in all cases. The use of negative suction drains over the mesh to eliminate the formation of seroma, or any collection in large hernia repairs has a very positive outcome and prevents postoperative and long-term complications. The placement of mesh in the retrorectus plane separates the mesh from both potential sites of bacterial translocation, i.e., the deep peritoneal cavity and subcutaneous space, as well as having less chance of recurrence. Mesh fixation in crucial places is of utmost importance to prevent mesh folding, migration, seroma, hematoma, and abscess formation. In retrorectus mesh fixation, we used Prolene 2-0 interrupted fixation to the posterior sheath meticulously, which is also a good way to fix the mesh, avoiding the full thickness fascial sutures, as described in classical Rives-Stoppa repair and hence less postoperative pain and faster recovery.

## References

[1] Lichtenstein IL, Shulman AG, Amid PK, Montllor MM (1989). The tension-free hernioplasty. Am J Surg.

[2] Mann DV, Prout J, Havranek E, Gould S, Darzi A (1998). Late-onset deep prosthetic infection following mesh repair of inguinal hernia. Am J Surg.

[3] Chen T, Zhang YH, Wang HL, Chen W, Wang J (2016). Late-onset deep mesh infection: a study of eight cases detected from 2666 consecutive patients with abdominal wall hernia repairs. Chin Med J (Engl).

[4] Falagas ME, Kasiakou SK (2005). Mesh-related infections after hernia repair surgery. Clin Microbiol Infect.

[5] Erdas E, Medas F, Pisano G, Nicolosi A, Calò PG (2016). Antibiotic prophylaxis for open mesh repair of groin hernia: systematic review and meta-analysis. Hernia.

[6] Rilna P, Guna TP, Joseph N, Raghu K (2019). Role of antibiotics in orofacial antibioma and its management: a case report. J Sci Dent.

[7] Waldrep DJ, Shabot MM, Hiatt JR (1993). Mature fibrous cyst formation after Marlex mesh ventral herniorrhaphy: a newly described pathologic entity. Am Surg.

[8] Ielpo B, Cabeza J, Jimenez D, Delgado I, Torres AJ (2011). Abdominal pseudocyst complicating incisional hernia repair: our experience and literature review. Hernia.

[9] Ogunbiyi SO, Morris-Stiff G, Sheridan WG (2004). Giant mature cyst formation following mesh repair of hernias: an underreported complication?. Hernia.

[10] Arya N, Batey NR (1998). Pseudocyst formation after mesh repair of incisional hernia. J R Soc Med.

[11] Mayagoitia JC, Almaraz A, Díaz C (2006). Two cases of cystic seroma following mesh incisional hernia repair. Hernia.

[12] Mehrotra PK, Ramachandran CS, Goel D, Arora V (2006). Giant pseudocyst of the anterior abdominal wall following mesh repair of incisional hernia: a rare complication managed laparoscopically. Hernia.

[13] Sahin-Tóth G, Halász T, Viczián C, Oláh T (2007). Late complication after mesh repair of incisional hernias: pseudocyst formation. Magy Seb.

[14] Narayanan CD, Dinesh N (2008). Abdominal wall pseudocyst following abdominoplasty after postmastectomy radiation therapy. Hernia.

[15] Hoefkens MF, Vles WJ (2008). A giant pseudocyst following repair of an incisional hernia by a propylene mesh. Ned Tijdschr Geneeskd.

[16] Mavros MN, Athanasiou S, Alexiou VG, Mitsikostas PK, Peppas G, Falagas ME (2011). Risk factors for mesh-related infections after hernia repair surgery: a meta-analysis of cohort studies. World J Surg.

